# Role of surface microgeometries on electron escape probability and secondary electron yield of metal surfaces

**DOI:** 10.1038/s41598-019-57160-w

**Published:** 2020-01-14

**Authors:** D. Bajek, S. Wackerow, D. A. Zanin, L. Baudin, K. Bogdanowicz, E. Garcia-Tabares Valdivieso, S. Calatroni, B. Di Girolamo, M. Sitko, M. Himmerlich, M. Taborelli, P. Chiggiato, A. Abdolvand

**Affiliations:** 1 School of Science & Engineering, University of Dundee, Dundee, Scotland UK; 2 CERN, European Organization for Nuclear Research, 1211 Meyrin, Switzerland

**Keywords:** Surface patterning, Surface patterning, Surface patterning, Experimental particle physics

## Abstract

The influence of microgeometries on the Secondary Electron Yield (SEY) of surfaces is investigated. Laser written structures of different aspect ratio (height to width) on a copper surface tuned the SEY of the surface and reduced its value to less than unity. The aspect ratio of microstructures was methodically controlled by varying the laser parameters. The results obtained corroborate a recent theoretical model of SEY reduction as a function of the aspect ratio of microstructures. Nanostructures - which are formed inside the microstructures during the interaction with the laser beam - provided further reduction in SEY comparable to that obtained in the simulation of structures which were coated with an absorptive layer suppressing secondary electron emission.

## Introduction

**Figure 1:**
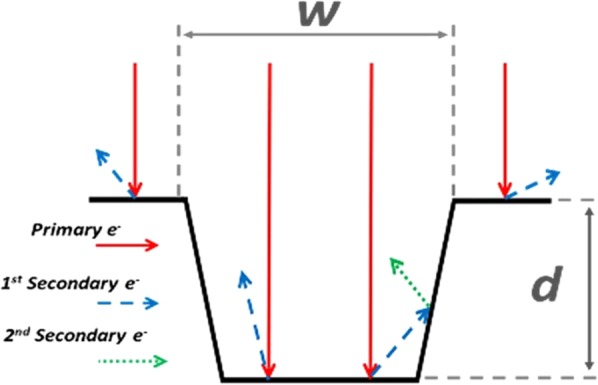
Primary electrons impinging on a trench within a flat metal surface leads to secondary emissions, which, upon colliding with the inner surface, only contribute to Secondary Electron Yield (SEY) if they have sufficient energy.

Secondary electron emission^1^ occurs for materials when a primary electron impinges upon the surface and induces successive excitation processes or is scattered. The number of emitted secondary electrons per primary electron is the Secondary Electron Yield (SEY). The surface topography and electronic properties of a material define the number of emitted secondary electrons, but also their ability to escape the structure. If the surface of the material is textured or otherwise non-flat, the emitted secondary electrons may go on to impinge upon a surface protrusion, leading to 2^nd^ generation secondary electrons. Since most of the secondary electrons are slow (a few eV of kinetic energy) and for metals the SEY of slow electrons is well below 1, only a few electrons of the second generation are extracted. Thus, the scattering on the surface protrusions results in a reduction of the total SEY compared to that of a flat surface, (see Fig. 1).

Whilst the process of generating secondary electrons has its advantages, for example in amplifying the desired signal in photomultiplier devices^2^, it is also cause for concern in a number of fields, from charging in spacecrafts^3^ to the limitation of stable beam current by electron cloud generation in proton accelerators^4^. As such, the development of methods to suppress electron cloud build-up is increasingly appealing^5^. The Large Hadron Collider (LHC) at CERN, for example, is a particle accelerator with a 27-km circumference^6^ in which vacuum pipe beam-screens are placed mainly to shield the cryogenic superconducting magnets from generated heat loads. These beam screens are made from co-laminated copper on stainless steel, which, in its as-received state results in a secondary electron yield well above 1, as for most air exposed metal surfaces. For instance, previous measurements of as-received copper resulted in SEY value close to 2^7^. In combination with the positive charge of the circulating proton beam, this is sufficient to provoke multiplication of the electrons in the vacuum beam pipe and generate the electron-cloud.

A number of SEY-reducing methods have been developed, including surface texturing, surface roughness and treatments of various materials^5,8,9^, deposition of porous structures on material surfaces^10^, modifying the surface topography by electrical discharge machining^11^ as well as Ti–Zr–V non-evaporable getter^12^ or amorphous carbon coating of the interior of the beam pipes^13^. Previously we have shown that laser-induced formation of microgeometries on metal significantly increases the optical absorbance of metal surfaces^14^, and furthermore reduce the SEY to close to 1^7^. The performance of these surfaces, which can exhibit a maximum SEY below 1, was successfully demonstrated in a test dipole magnet and in a cryogenic straight in the Super Proton Synchrotron (SPS) accelerator at CERN^15,16^.

In this paper, we report on an experimental demonstration of how the dimensions of the structures created through laser-metal interaction affect the electron escape probability and therefore the SEY of copper, where tightly positioned parallel grooves are carved into the surface whose dimensions may be controlled by varying the laser parameters. The following experimental results are compared with similar simulations by Ye *et al*.^17^. The simulations – based on assuming perfect rectangular grooves - predicted that the escape probability *P*_*e*_ of an electron impinging the bottom-centre of the trench at normal incidence would fall with increasing aspect ratio *A*_*s*_ (that is, the ratio of height to width of the trench) according to:

$${P}_{e}=\,\sin (\arctan (2{A}_{s}{)}^{-1})$$

The formula describes effects in the trenches and the resulting total SEY is obtained by a weighted average of the trench region and the remaining flat regions. From this model it was predicted that an increase in aspect ratio (height to width) would reduce the secondary electron yield. Beyond the influence of the aspect ratio on the SEY, the simulation also shows that if the trench’s inner surfaces are coated with an absorbing texture, which is simulated such that if it were a flat surface, it would ordinarily give rise to an SEY of unity, the escape probability (and therefore SEY) are further reduced^17^. The simulated results find that compared to a flat copper surface, the SEY will lower by approximately 19% when trenches of aspect ratio 1.0 are introduced, and by 38% when the trenches are coated. Similar Monte Carlo simulations have recently confirmed this theory^18^.

The experimental observations presented here can be interpreted accordingly as a laser engineered surface with trenches leading to a reduction in SEY superimposed with nanostructures created along the inner surface of the trenches, which are comparable with an absorptive coating, further reducing the SEY. We demonstrate this by testing the SEY of copper samples before and after these nanostructures are removed in a rigorous cleaning process.

## Methods

### Creating laser-treated copper samples

Six identical unprocessed copper samples of equal dimensions (20 mm long, 15 mm wide and 2 mm thick) were prepared for laser structuring by firstly washing in isopropyl alcohol and drying in order to remove residues. These were individually placed on an adjustable 3-directional stage situated underneath a software-controlled 2-dimensional scan head equipped with a tele-centric lens. A solid-state pulsed laser beam operating at 532 nm wavelength, 200 kHz repetition rate and 10 picosecond pulse duration was coupled into the optical scan head, and the 3-dimensional stage was adjusted such that the upper surface of the copper sample was positioned at the focal length of the tele-centric lens. This focal position was adjusted for a nitrogen-filled glass chamber which was placed over the sample in order to supress oxidation of the copper during laser structuring.

**Figure 2:**
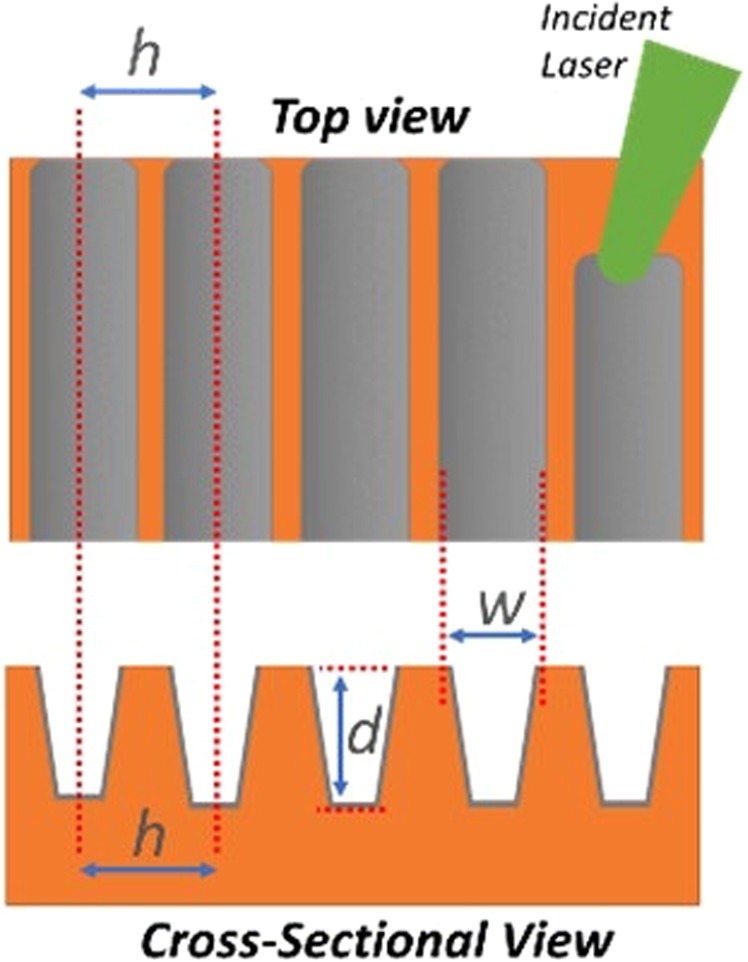
Diagram illustrating the laser treatment pattern on the copper surface (top) and cross section (bottom) where *h* is the hatch distance between laser treatment lines, and *d* and *w* are the resulting depths and widths of the trenches created whose aspect ratio (d/w) reduces the electron escape probability.

After copper sample preparation, the laser beam was then attenuated remotely such that an average fluence of approximately 1.9 J cm^−2^ (peak fluence of 3.8 J cm^−2^) was incident upon the copper surface. The experimentally measured value for the ablation threshold of copper at 532 nm was ~0.24 J cm^−2^ (peak fluence). The assessed thermal threshold of copper at 532 nm was ~10.6 J cm^−2^. These values were experimentally determined through the self-consistent evaluation of the Gaussian beam diameter and the ablation threshold^19^. A scanning pattern was programmed via the software control to the scan head such that individual parallel grooves may be written across the surface, at a set width apart (known as the hatch distance *h*), as shown in Fig. 2. For this study, the laser beam scanned the surface at 5 mm/s, 10 mm/s, 15 mm/s, 20 mm/s, 25 mm/s and 30 mm/s for six samples, resulting in the delivery of 2080, 1040, 693, 520, 416 and 347 pulses per individual spot on each sample, respectively. In each case a 15 mm × 15 mm area (225 mm^2^) of the copper surface was laser processed in total, made up of parallel lines spaced apart by a hatch distance *h* of 91 µm. This hatch distance was selected to allow an optimal distance between lines such that no overlapping of lines would occur for accurate comparison with the theoretical predictions.

### Measuring the SEY of copper samples

SEY measurements of the samples were performed in UHV using the experimental setup that was described in detail elsewhere^20^. The samples were inserted in the measurement chamber using a load-lock system. The measurements were performed by scanning the energy of the primary electrons that impinge on the surface at normal incidence in the range from 50 to 1750 eV, and simultaneously measuring the currents to the sample and to the secondary electron collector. The collected data allowed for the SEY to be calculated. SEY measurements were performed on three different spots of about 2 mm^2^ in order to assess reproducibility of the treatment. The total dose that is delivered during the measurement of each SEY curve is ~3 × 10^−7^ C mm^−2^. This value is low enough to prevent conditioning effects during the measurements. It is worth pointing out that no surface charging was detected during the measurements, and the measurement curve was reproducible within the experimental uncertainty (estimated at 0.05).

### Removing nanostructures on the inner surfaces of the trenches

Each sample was fixed on one edge by a crocodile clamp and upright fully immersed into an 80 mL beaker filled with ultra-pure water. The beakers were placed into an ultrasonic bath at ambient temperature. Two cycles of 100 min were applied at the intensity of 300 W and frequency of 45 kHz. The efficiency of the ultrasonic cleaning seemed to depend on the immersion depth. Therefore, the cleaning cycles were repeated after turning the samples by 180 degrees in order to homogenize the cleaning across the sample surface. The samples were weighted before and after the ultrasonic cleaning process to assess the quantity of nanostructures detached from the surface. XPS analysis did not reveal major changes in the surface composition upon cleaning. The extracted particles were collected by filtration of the particle-containing water using an isopore (the size of the pores was ~500 nm), polycarbonate, hydrophilic membrane. The collected particles were weighted as well. According to the weight-loss analysis, on average 93% of the detached nanostructures were collected in the membrane. A linear correlation between the number of laser pulses during the treatment and the amount of detached particles during ultrasonic cleaning was found.

### Top-view microscopy and cross-section analysis

Scanning electron microscopy and optical microscopy were used to investigate the surface aspect ratio and cross-section of the samples. Top view images were measured by a FEG Sigma SEM (ZEISS) equipped with a field-emission source and an in-lens as well as an Evan-Thornley detector for Secondary Electron imaging. Several regions of each sample were analysed.

To prepare cross-sections, samples were mounted vertically in a transparent resin with metal supports. After mounting, the samples were ground with SiC papers down to grit P1200 and polished with diamond suspensions gradually reducing the particle size from 3 µm to 1/4 µm. Cross-Section images and dimension analysis was performed using a KEYENCE VHX 6000 optical digital microscope.

For simplification of comparison, the aspect ratio as defined in this work is the same as that given in the theoretical model by Ye *et al*.^17^, i.e. trench depth *d* to trench width *w*, or simply *d/w*.

## Results

**Figure 3:**
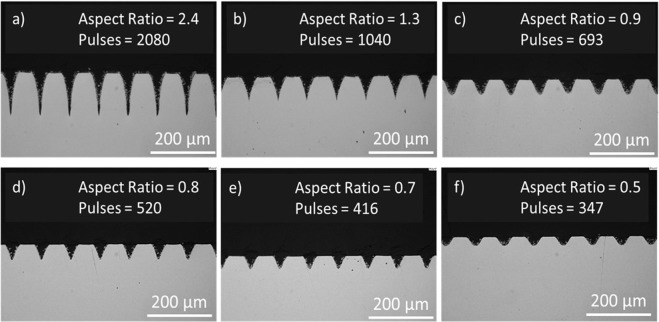
Cross-sectional optical images of the structured surfaces of each sample shows a decreasing trench depth for decreasing number pulses delivered at the interaction spots. Digital measurements of each trench depth and width provided values for decreasing trench aspect ratio (**a–f**).

The laser-structured samples were characterized by both Scanning Electron Microscopy (SEM) and optical microscopy in order to measure the depths and widths of the microstructures created, see Fig. 3. Faster beam scanning rates (delivery of fewer pulses per interaction spot) clearly resulted in shallower penetration depths, whereas the trench widths appeared to remain constant. This effect was confirmed by trench analyses of multiple sample regions.

**Figure 4:**
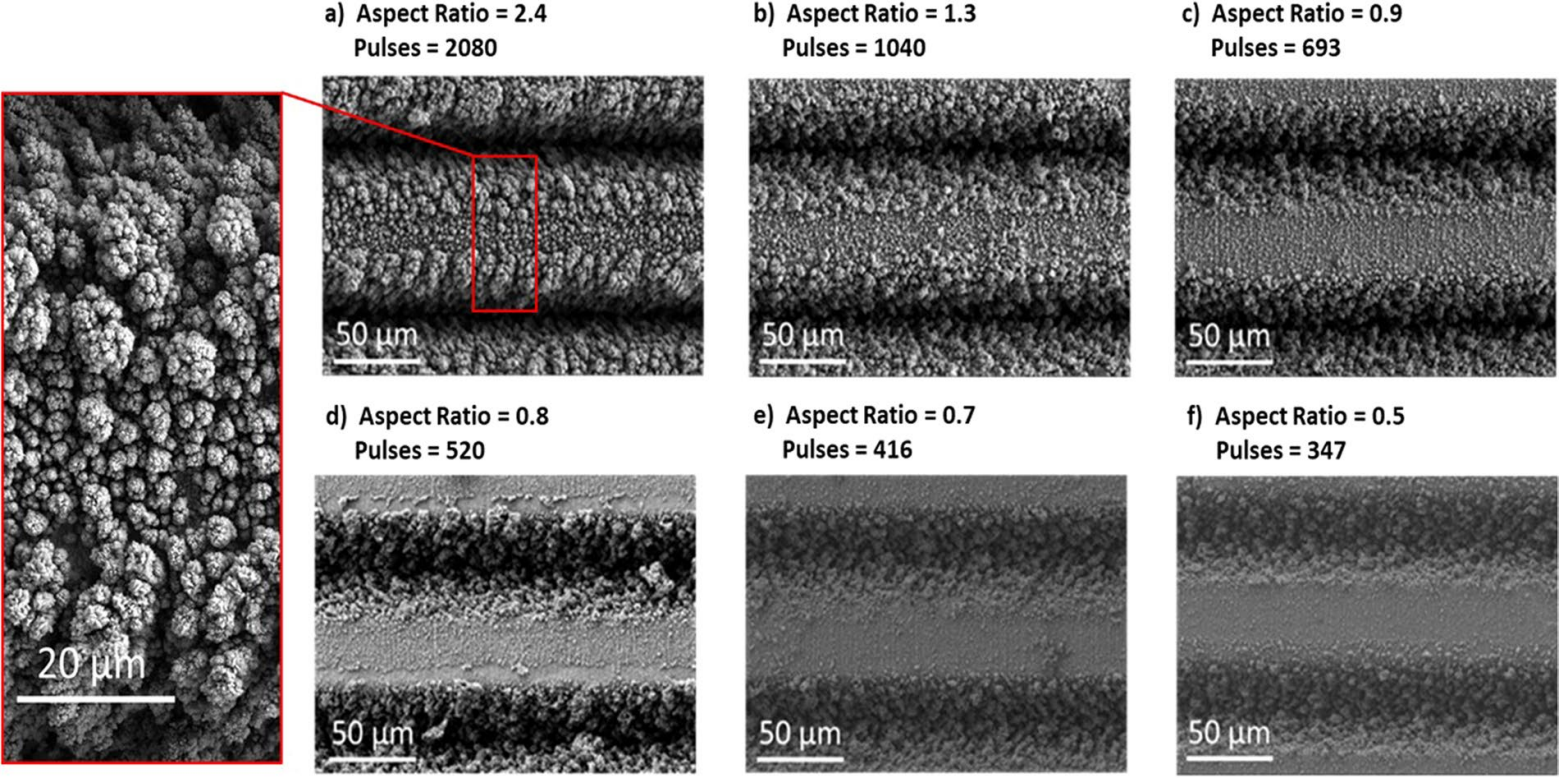
Top surface SEM images of the samples before cleaning for decreasing aspect ratio (**a–f**). Highlighted region, showing the nanostructures which are created during processing and effectively coat the trenches.

The trench depths decreased on average as 126 µm, 70 µm, 49 µm, 43 µm, 37 µm and 27 µm respectively due to the decreasing pulses per spot interacting with the surface, whilst the trench widths remained close to 52 µm due to the constant laser beam fluence incident on the samples. Moreover, since the hatch distance also remained constant, the macro geometry (spacing between grooves) also remained constant, therefore in the following analysis, only the aspect ratios of the trenches are considered. It was therefore demonstrated that an increase in laser pulses per spot gave rise to an increase in trench aspect ratio. Top-view scanning electron micrographs of the trenches and the existing nanostructures at the surface after laser structuring (without cleaning process) are shown in Fig. 4.

**Figure 5:**
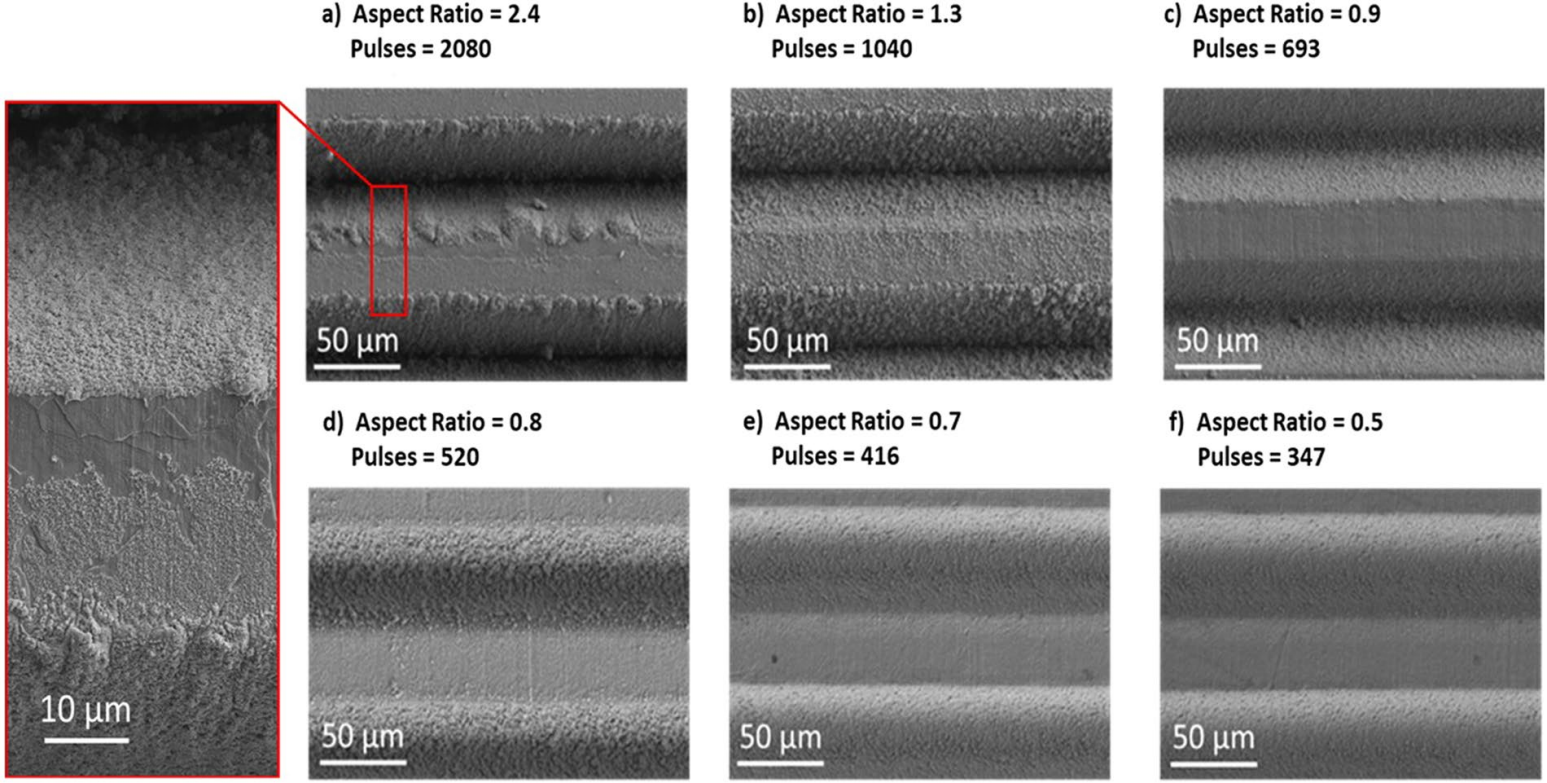
Top surface SEM images of the samples after cleaning, showing the removal of the nanostructure coatings for decreasing aspect ratio (**a–f**).

After each sample had undergone a cleaning process to remove the layer of nanostructures, SEM imaging of the same regions were repeated, see Fig. 5.

**Figure 6:**
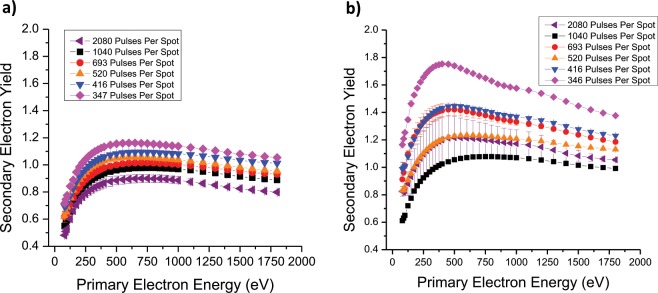
The measured SEY traces as a function of laser paramteres. (**a**) Before the samples were cleaned of nanostructures within the grooves, and (**b**) after the nanostructures were cleaned, leaving bare trenches.

The SEY of each sample was then tested and plotted as a function of laser parameters, both before and after the removal of the nanostructures which coat the inner surfaces of the trenches, see Fig. 6.

**Figure 7:**
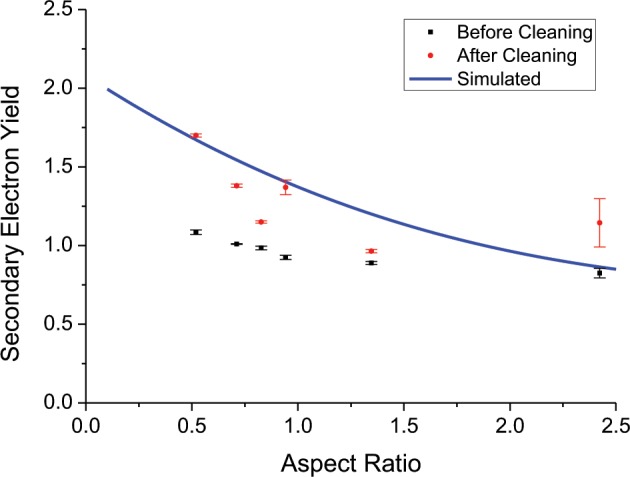
The measured maximum average SEY from each sample both before and after the removal of the nanostructures within the trenches, plotted as a function of the measured trench aspect ratio, and compared with the simulated trend. The primary electron energy is 300 eV.

For direct comparison with existing simulation results^17^, experimentally measured average SEY values at 300 eV primary electron energy were extracted for each curve and plotted versus the measured aspect ratio, together with the simulated values, see Fig. 7.

It is well documented that the composition of the respective copper surface has an influence on the secondary electron yield^21^. In order to define the surface stoichiometry of the samples under consideration in this manuscript, their surface composition was analysed by X-ray photoelectron spectroscopy using MgKα radiation. The surfaces created after laser structuring are slightly oxidized and nitrogen was incorporated into the top surfaces and is considered to be a technical surface, not free of impurities. Furthermore, a slight uptake of ambient adsorbates is evident from the measured XPS spectra that are shown in the supporting information, see supplementary information, Fig. S1. Assuming homogeneous elemental distributions, the surface stoichiometry for the laser-treated (ultrasonically cleaned) samples was determined to be: 38.8 (28.5) at. % copper, 41.1 (28.5) at. % oxygen, 7.8 (5.3) at. % nitrogen, 12.3 (37.7) at. % carbon. The individual core level spectra did not indicate any significant spectral change - only the amount of surface carbon impurities slightly changed after the ultrasonic water cleaning. This indicates that the main changes in SEY are due to topography changes rather than chemical surface changes.

## Discussion

The trend of the experimental values shows a moderate decrease of the SEY at 300 eV primary electron energy as a function of the aspect ratio. Therefore, the laser treatment can modify the SEY in a controlled manner. The same trend is reflected in the simulation. The samples without nanostructures exhibit systematically higher SEY (Fig. 6) showing that the decrease of the maximum SEY below unity is given by the combined contribution of the trenches and nano-structures. The removal of the nanostructures also lowers the maximum energy of the SEY curve (Fig. 6), which is an indication of reduction of the roughness. Moreover, the samples without nanostructures exhibit a stronger decrease of SEY as a function of aspect ratio. This is understood by the fact that the SEY of as-treated samples is dominated by the nanostructures and the aspect ratio dependence is thus somehow weakened. Discrepancies in the values between experiment and simulation may be due to several reasons. We must consider firstly that the theoretical values in^17^ are based primarily on a simplified scenario of a perfectly square trench, whereas trenches created in practice were either square-rounded, or triangular-rounded, and thus the re-direction of secondary electrons will vary due to a variation in the model of electron escape probability. Additionally, we have shown previously using SEM^15^ that the trenches of laser-treated copper samples are not perfectly smooth like the simulated scenario above, but rather they are composed of random, tightly packed nanostructures of varying dimensions (see Fig. 4, highlighted region). It is thought that this feature acts like a coating of the trench, and further reduces the SEY. A similar theoretical observation was reported by Ye *et al*.^17^ who alongside an uncoated trench system, simulated a coated trench system with an electron absorbing layer.

Furthermore, the simulated results showed around a 19% decrease in SEY maximum due to the addition of the absorptive coating layer on the trenches, and the experimental counterpart results showed an average decrease of a similar magnitude due to the presence of nanoparticles in the trenches (compared to after they are removed by cleaning). The comparison cannot be extended further, since the shape of the SEY curve as a function of energy in the simulation and experiment are different due to the arguments mentioned above. In addition, the total SEY of our samples is influenced by the flat regions between the trenches, a further approximation compared to the simulated model, resulting in an offset of the measured SEY towards higher values.

In conclusion, by comparing the experimental and theoretical microgeometries we demonstrated that an increased aspect ratio of the structures formed by laser structuring on the surface of copper leads to a decreased SEY, due to the reduced electron escape probability. This was achieved by changing the number of laser pulses delivered per interaction spot on the copper surface, leading to the increase in the aspect ratio of the microgeometries due to an increase in their depth, and thus reducing the SEY to below *unity*, at approximately 0.9. These experimental results were compared with those found by simulation and are in good agreement with the predicted trend. The nature of laser-surface interaction, which leads to the formation of complex nanostructures on the trench’s inner surfaces, act like an absorptive coating, allowing further suppression of secondary electron emission, and supports the studies in this work.

## Supplementary information

Supplementary Information.

## Data Availability

The datasets generated during and/or analysed during the current study are available from the corresponding author on request.
