# Supplementary material for: Role of surface microgeometries on electron escape probability and secondary electron yield of metal surfaces
Source: Sci Rep. 2020 Jan 14;10:250. doi: 10.1038/s41598-019-57160-w (PMC6959246; doi:10.1038/s41598-019-57160-w)
Supplement: Supplementary file 1 — Supplementary Information. [file 41598_2019_57160_MOESM1_ESM.pdf]

Role of surface microgeometries on electron escape probability and secondary electron yield of metal surfaces

D. Bajek [1], S. Wackerow [1], D. A. Zanin [2], L. Baudin [2], K. Bogdanowicz [2], E. Garcia-Tabares Valdivieso [2], S. Calatroni [2], B. Di Girolamo [2], M. Sitko [2], M. Himmerlich [2], M. Taborelli [2], P. Chiggiato [2], A. Abdolvand [1 †]

[1] School of Science & Engineering, University of Dundee, Dundee, Scotland, UK

[2] CERN, European Organization for Nuclear Research, 1211 Meyrin, Switzerland

[†]a.abdolvand@dundee.ac.uk

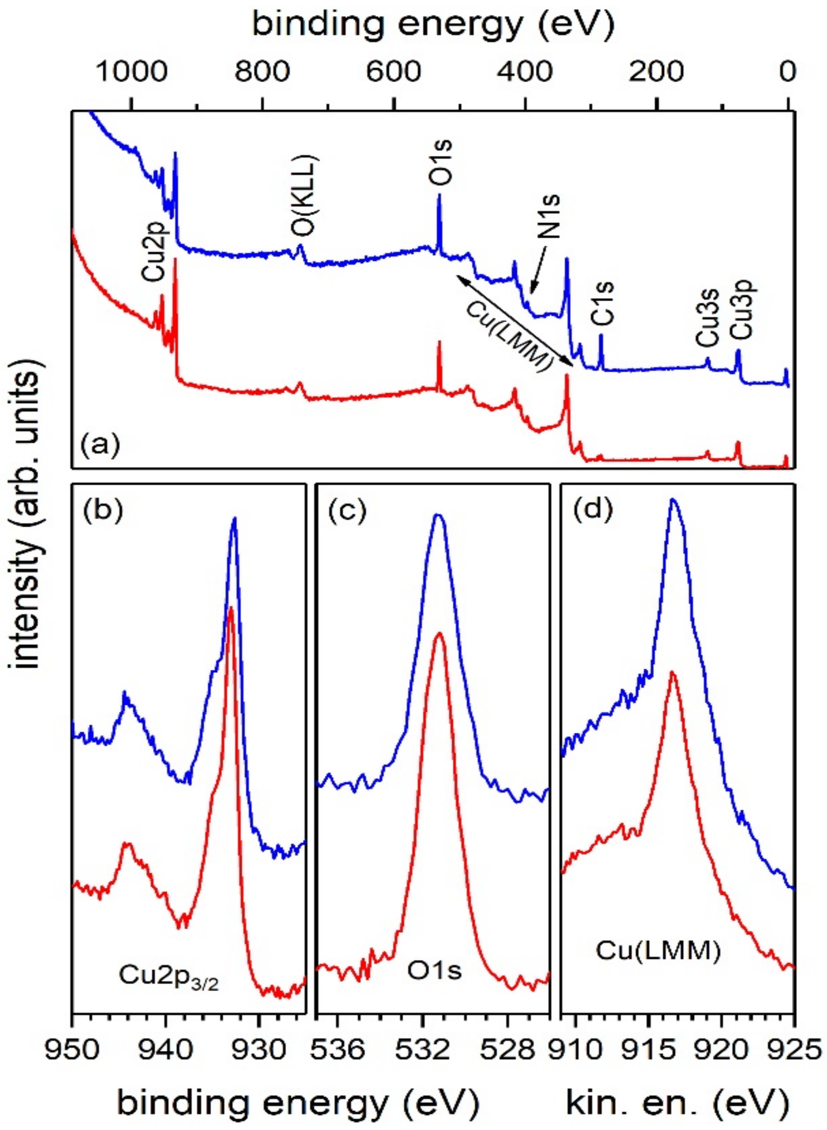

Supplementary Figure S1: X-ray photoelectron survey (a) main core level (b)-(d) spectra of the laser-treated Cu surface before (red) and after ultrasonic cleaning (blue).
